# The importance of the social environment on leisure destination choice: A mixed multinomial analysis of homophilic preferences

**DOI:** 10.1177/23998083241303198

**Published:** 2024-11-29

**Authors:** Benjamin Gramsch-Calvo, Kay W Axhausen

**Affiliations:** 111840 ETH Zurich, Switzerland

**Keywords:** Leisure destination choice, social environment, leisure segregation, self-selection, activity-based segregation

## Abstract

Individuals are fond of belonging to a social environment with a similar social background, which can impact the individual’s decision to visit specific venues for leisure activities. Using data from Zurich, we have measured the preference for a social environment in four categories of leisure venues: restaurants, cafes, bars, and nightclubs; the estimation was performed using a mixed multinomial logit model to see how homophily for socioeconomic characteristics can impact the decisions of choosing a leisure venue. The models included three homophilic preferences: age, income, and cultural origin as variables of interest. The results show a positive impact of the three variables in different degrees: age is the most relevant in the two venue categories, income only impacts when individuals choose restaurants or cafes, and cultural background is more relevant for nightlife venues. These results show that the sociodemographic characteristics of the social environment are relevant for the choice of leisure destinations. These findings can contribute to the formulation of policies to create more diverse leisure environments and socially cohesive communities.

## Introduction

Leisure travel is an essential part of urban mobility, accounting for 43% of trips in Switzerland (Bundesamt für Statistik, 2023b), with increasing importance over the last decades. One difference between leisure travel and commuting is its heterogeneity in motivations, schedules, and routines; leisure travel has less rigid spatial and temporal constraints, giving more freedom to the individual to perform it at different times and places (Schlich et al., 2004). Regardless of this heterogeneity of motivations, one common characteristic is its social motivation, since most of these activities are carried out in pairs or groups to maintain social connections or expand one’s social network (Axhausen, 2005, 2008). The two main types of social motivations studied to understand leisure travel have been the ego-alter and the ego-network. Ego-alter analysis is the study of the relationship between an individual (ego) and a friend, family member, or other acquaintance (alter) of the ego, focusing on the sociodemographic characteristics of the individuals, their relationship status, frequency of communication and emotional closeness, and is used to understand how specific individuals belonging to the ego’s social network impact the ego’s travel behavior. Ego-network analysis focuses on the overall characteristics of the ego social network, such as the number of friends and family members, their distribution in space, and the resources available from the network; this analysis helps to understand the macro-level influences that networks have on the egos (Carrasco and Miller, 2009). In this paper, we propose a third type of social motivation for social-leisure travel: the social environment, which is the sociodemographic characteristics of a group of unknown individuals that share a common space, in this case an urban leisure venue. We base our hypothesis on the interest of individuals in belonging to a social context founded on shared experiences, beliefs, or personal characteristics (Mahar et al., 2013), which can be a primary motivation for leisure. However, preferences for venues with specific social environments can lead to self-selection into those venues, contributing to an overall increase in urban segregation.

Since the mid-1970s, segregation has been on the rise in many countries, and understanding its causes and effects has become an important topic of interest in urban studies (Musterd et al., 2017). Segregation is a complex spatial phenomenon, as it responds to multiple factors, including social, economic, and political, and cannot be reduced to purely residential clusters, as residential segregation does not necessarily imply social segregation (Vaughan and Arbaci, 2011). The use of public and semi-public spaces for leisure can have a large impact on interactions between different social groups in daily life, as leisure spaces have replaced firms and workplaces as organizing units in society (Florida, 2003), becoming an essential contribution to the social sustainability of cities and a source of face-to-face interaction between unknown people, generating a sense of community (Jacobs, 1961; Nasar and Julian, 1995) and improving the general quality of life of the population (Bramley and Power, 2009). Therefore, understanding the importance of self-selection for the social environment in leisure activities can help create more socially diverse venues to help heterogeneous social interactions thrive.

To test the importance of the social environment in the leisure destination choice process, we have conducted a survey in Zurich, Switzerland, asking about regularly visited leisure venues. Later, a mixed multinomial logit was estimated; this model measures the impact that homophily, as our variable of interest, has on the decision to go to a venue. Homophilic preferences are multifactorial, with age, income, education, race, and religion being the most important sociodemographic characteristics of tie formations (McPherson et al., 2001). In this paper, we focus on three characteristics of the social environment: age, income, and cultural origin. These preferences are studied in four types of leisure venues: restaurants, cafes, bars, and nightclubs. The models show that the three homophily variables studied positively and significantly impact the decision to choose these venues for leisure activities and can be relevant to understanding how segregation dynamics are generated in daily activities.

## Literature review

Activity-based segregation has been a topic of growing interest in recent years. A new concept that has been applied to understand the phenomena is the *multi-contextual segregation*, defined as the uneven spatio-temporal distribution of daily life and the uses of the city of different social groups; this definition integrates time and space components into a conceptual framework, improving the understanding of segregation based only on space (Park and Kwan, 2018). Differences in time-space uses of the city can be generated by differences in income, activity characteristics, and the built environment, as shown by Wang et al. (2022). The most affected social group is lower-income individuals as they are highly dependent on the areas around their homes to carry out leisure and shopping activities; these spatial boundaries are not due to geographical constraints but to economic affordability (Zhang et al., 2019). For example, in Brazil, high-income white people have the highest access to leisure activities due to the concentration of venues around the neighborhoods where this group lives and the higher access to motorized private transport; while low-income black populations live further away from leisure venues and have less access to mobility tools (Bogado Tomasiello and Giannotti, 2023). Wu et al. (2023) have studied the gathering capacity of different areas of Shenzhen, showing that high-tech areas of the city have a high gathering capacity for heterogeneous populations, while in terms of specific venues, food and beverage services vary in their capacity to gather individuals from different socioeconomic backgrounds, as consumption habits vary significantly between individuals. The results align with studies on the ethnolinguistic co-presence of individuals, showing that the city center can be an essential space to interact with culturally different individuals (Toomet et al., 2015). These studies have focused on how the built environment and accessibility can impact the segregation of leisure activities. However, to date, there is still a need to understand the behavioral mechanisms that can explain activity-space segregation. A first endeavor into this topic was made by Chen et al. (2024), who studied how individual choices affect activity-space segregation, showing that people with medium and high education levels prefer destinations with mixed social environments. Still, more research is still needed to dive deeper into the behavioral motivations that can generate activity-based segregation. For this reason, our interest is to analyze the behavioral origins of segregation with a focus on leisure activities.

To study leisure behavior, the literature has focused on the social context and the need for interaction with other individuals. In order to understand travel decisions for social activities, the main focus has been on ego-alter and ego-network characteristics (Kim et al., 2018). In terms of ego-alter characteristics, the models of social activity participation have estimated different parameters that impact the generation of face-to-face interaction, such as the distance between individuals (Van Den Berg et al., 2010) and the type of relationship (Carrasco and Miller, 2009). Han et al. (2023) has focused on the impact of cliques on restaurant choice, showing that cliques tend to choose venues based on the mean distance to the venue of individuals who belong to that clique. The social influence of travel affects the overall individual’s mobility patterns; individuals with a more dispersed social network also generate a more dispersed leisure activity space (Gramsch-Calvo and Axhausen, 2024a). Furthermore, individuals with more extensive social networks tend to have greater heterogeneity in the type of sites visited and to perform more socially motivated travel (Baburajan, 2019).

In social networks, a recurring topic is homophily; individuals tend to interact and create connections with other similar individuals, creating personal networks that are homogeneous in terms of sociodemographic, behavioral, and intrapersonal characteristics, generating clusters in social space (McPherson et al., 2001). Homophily has been studied for different aspects of social life, such as housing markets (Galster et al., 2021; Pinchak et al., 2021), friendship (Currarini et al., 2009), and migrant communities (Vacca et al., 2022), and can have positive and negative effects on social systems; on one hand, it promotes cooperation between individuals and diffusion of knowledge (Korkmaz et al., 2020; Melamed et al., 2020) but on the other hand, it can generate social segregation (Schelling, 1971).

The decision to conduct leisure in a specific location can be influenced by a wide variety of temporal, spatial, personal, and social factors, making it almost impossible to estimate choice models that can be generalizable to all choice situations, generating a variety of approaches, methodologies, and theoretical structures adjusted for the context of the models (Barnard, 1987). One of the most common methodologies is the multinomial logit model (McFadden, 1973), which can individually disaggregate the alternatives in the choice set. Despite this benefit, there are still challenges associated with destination choice models that are not directly related to Random Utility Theory, but to the data used to estimate them. Most research using this technique still uses zonal-level characteristics, such as the number of venues, population, and OD impedance variables of interest (Wang and Miller, 2014), which can be misleading for low-density areas (Molloy and Moeckel, 2017). To improve the information about the choice process, researchers have started using social networks to collect data on variables of interest, allowing them to increase the available information (Rashidi et al., 2017); however, these models still rely primarily on zonal characteristics. In this paper, we combine data collected using a survey with venue data collected from a Social Network Service (Google Places) to understand the choice process at the venue level, improving the level of detail of the models by using information that was previously expensive to collect, such as prices and ratings of the venues studied.

Urban leisure segregation has been studied mainly as the co-presence of dissimilar individuals in the same areas of a city. However, this is not sufficient to ensure the mixing of social groups, as leisure activities can be performed in adjacent venues with different social environments. For this reason, it is essential to integrate leisure behavior to understand how social travel can be influenced by the preference to be part of a specific homophilic social environment at the venue level, separating the segregation generated by individual behavior from the segregation generated by the built environment.

## Data description

To collect data for this study, a three-stage survey was designed and implemented in the Zurich Metropolitan Area, during November and December 2022. It contacted 8000 randomly selected addresses obtained from a private address provider. The invitation was sent by mail with a letter specifying the research, a link and QR code with the first stage of the survey, and a six-letter code for personal identification. After completion of the first stage, an invitation for the second stage was sent 3 days later by email, if provided by the participant, the third stage was sent 3 days after the second. There was a 15 CHF incentive (16.2 USD at the time of the survey) for respondents who completed the three stages of the survey. The analysis of this manuscript only uses the data collected in the second stage; therefore, the data description and survey design will focus on that stage.

The second stage of the survey was separated into two parts, the first part is called the *place generator* (Gramsch-Calvo and Axhausen, 2024b) which is a methodology adapted from the *name generator*, widely used in social network analysis; in this part, the respondent had to name the venues they visit on a regular basis in seven categories: restaurants and cafes; bars and nightclubs; parks, forests and other outdoor locations; cultural venues; social or religious centers; gyms and sport-centers; and others. Each category had up to three spaces that could be filled in with the names of the venues. The second part of the stage was the *place interpreter*, which consisted of a group of questions that were repeated for each of the venues mentioned previously; the questions were related to the visit schedules (i.e., regular visit time, days of the week, and frequency of visit), reasons to go, and characteristics of the place. The main question of interest in this section was to describe the sociodemographic characteristics that the respondent believes they have in common with the other visitors to the venue; this question was later used to reproduce the social environment of the venues included in the study.

The survey has 975 respondents, with 9721 venues mentioned. In terms of respondents, after cleaning individuals that did not mention their home location (for privacy reasons, this question was not mandatory), and individuals that did not mention any restaurant, cafe, bar or nightclub, there are 743 individuals included in the analysis. Table 1 shows the distribution of the main sociodemographic characteristics of the individuals included in the analysis, compared to the average population, the survey is representative in terms of gender, with a slightly higher proportion of males; for household income, our survey shows an average salary of 10,507 CHF monthly, while the Zurich average is 9780 CHF; with respect to origin 32% of the respondents are foreigners or second-generation immigrants versus 27.4% city-wide. In terms of age, our survey has a higher proportion of individuals in the range of 51–60 years old, making the average age 55 versus the average population of 48 (considering individuals over 18 years of age) (Bundesamt für Statistik, 2023a).

**Table 1.** Sociodemographic characteristics of surveyed individuals.

Gender	
Male	53%
Female	47%
Household income	
Under 2000 CHF	1%
2,000–4,000 CHF	2%
4,001–6,000 CHF	9%
6,001–8,000 CHF	15%
8,001–10,000 CHF	16%
10,001–12,000 CHF	17%
12,001–14,000 CHF	12%
14,001–16,000 CHF	8%
16,000–18,000 CHF	6%
More than 18,000 CHF	9%
I prefer not to say	5%
Age	
30 or less	3%
31–40	10%
41–50	20%
51–60	39%
61–70	28%
70 or more	0.1%
Origin	
Swiss	68%
Western Europe	22%
Eastern Europe	4%
Mixed	2%
Other	3%

In terms of venues, the focus of this manuscript is on restaurants, cafes, bars, and nightclubs; we have chosen restaurants and cafes because there is a large offer of venues and it is a widely popular leisure activity, while bars and nightclubs were chosen because of the social nature of the activity, being the possibility to interact with unknown people an essential component of the experience. Therefore, after selecting these types of venues, we had excluded answers that were too broad or not venues, and places that were not possible to geocode. Finishing with a total of 3207 observed choices from 743 individuals and 1653 different venues. As cafes and nightclubs do not have enough observations to be estimated separately, we have created two joint categories: *food venues* and *nightlife venues*. The grouping and details of each category are presented in Table 2.

**Table 2.** Categories used for the estimation.

Category	Number of venues^a^	Number of choice situations^b^	Grouped category
Restaurants	1174	1842	Food services
Cafés	204	354	
Bar	351	824	Nightlife
Nightclub	70	187	

^a^There are 1653 different venues, but the sum of this column is 1799. This is because some venues are part of two categories.

^b^The categories were combined for the estimation, but the choice sets were generated only with the same original category (i.e., nightclubs are considered under nightlife but their choice set is composed only other nightclubs).

**Figure 1.**
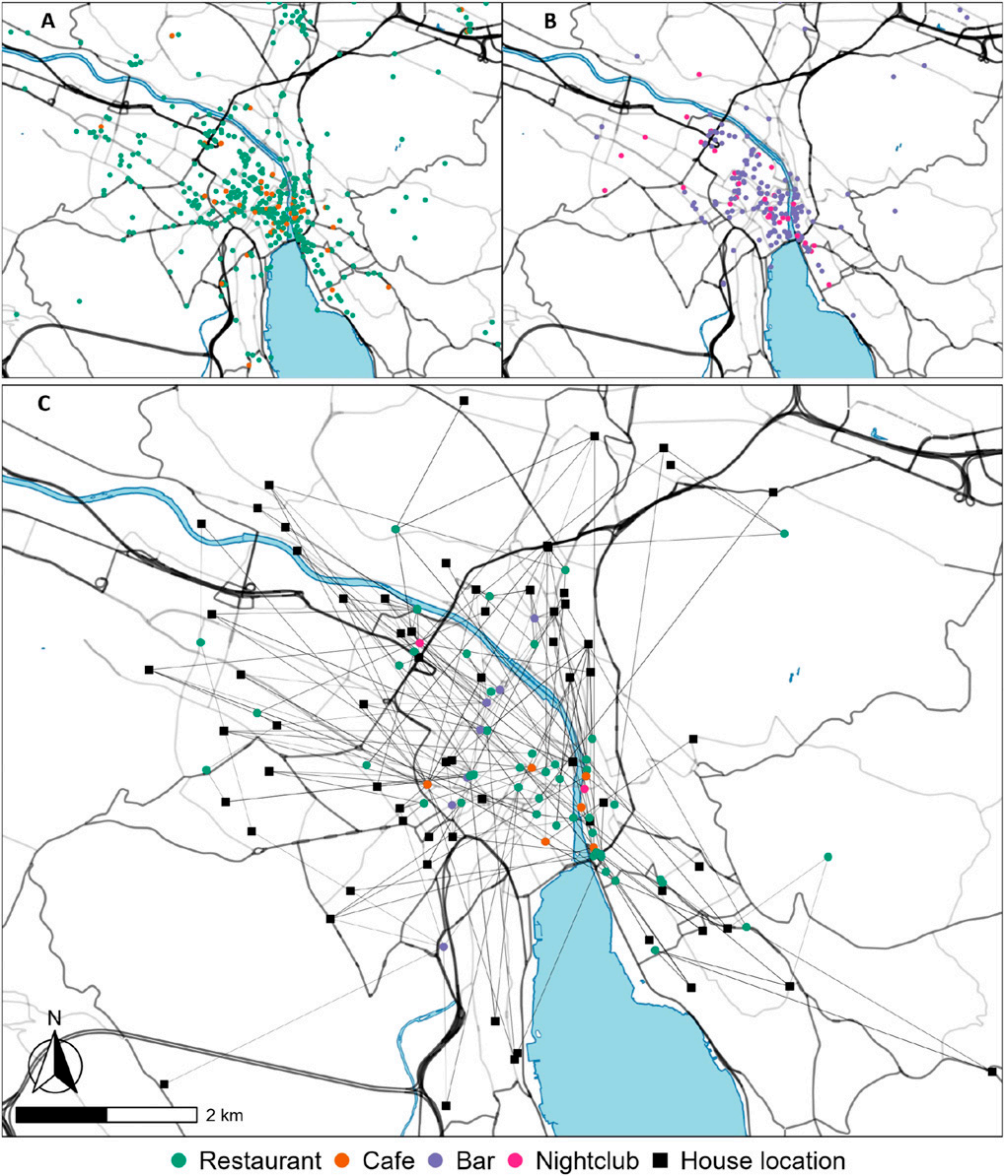
Sample of distribution of: (A) food venues, (B) nightlife venues, and (C) example social networks of a leisure location.

To obtain the geographic locations, categories, prices, and ratings of the venues, we geocoded the responses with the Google Places API (Cambon et al., 2021). The first information collected by geocoding is the spatial location of the venues. Figure 1 shows the distribution of the venues. Plot A is the spatial distribution of food venues in Zurich; plot B is for the nightlife venues. Plot C is a sample of the network formed by leisure activities, representing how unknown individuals (squares) are connected through leisure venues (circles). By participating in leisure activities, individuals generate the social environment of the venue visited, generating a complex network of interactions and communities.

**Figure 2.**
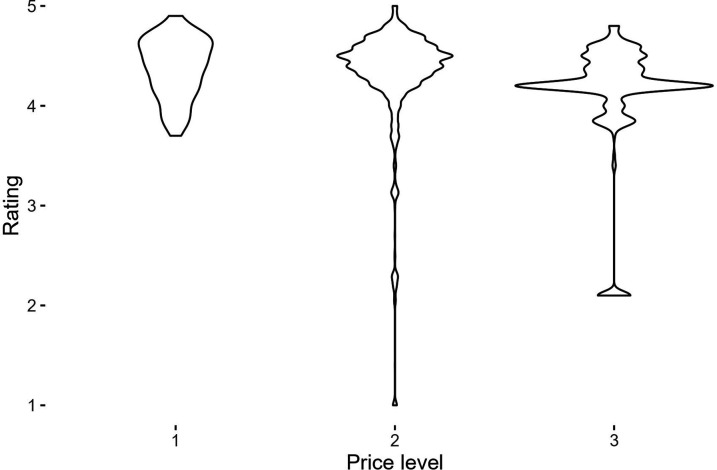
Relation between price and rating of the venues visited.

The second set of information obtained from the geocoding process used is the rating and price of the venue. This information, combined with the information provided by the respondents, creates a dataset of venues to analyze. The first analysis compares the rating with the price level of the venues. Figure 2 shows the relationship between these two variables. The rating variable ranges from 1 to 4.9, with most venues in the 4 to 5 range. Regarding the price levels, the variable ranges from 1 to 4. The price level is also a proxy for the type of service offered; restaurants price level 1 are primarily fast food and budget-focus restaurants, while restaurants price level 4 are fine dining; but due to the lack of available locations with price level 4 (10 venues), we have excluded them from the analysis. The correlation between rating and price is negative, but low (−0.08). As the rating is user-based, one could expect that individuals have rate venues depending on the price level and service expected. The distance was calculated as the Euclidean distance from the home location of the respondent to the venue; individuals live in a median distance of 2.9 km from their regular leisure venues.

**Figure 3.**
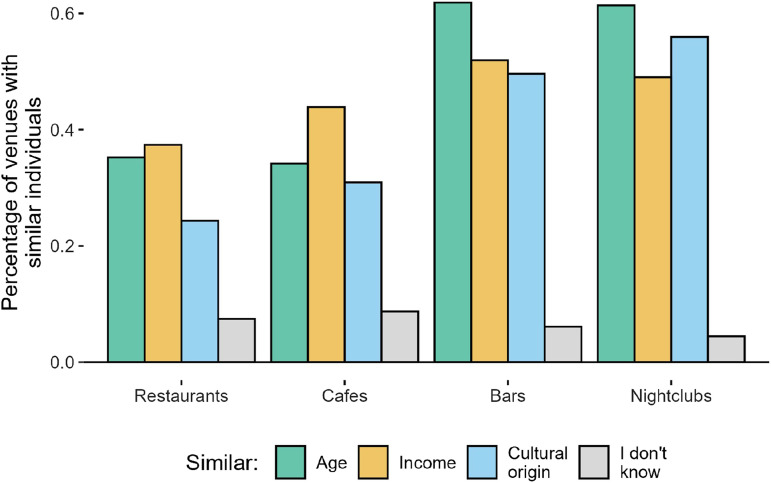
Homophily described by individuals.

To recreate the social environment of each venue, we have included a question about the type of visitors, in relation to the respondent, who frequent the venues mentioned. The answers to this question are presented in figure Figure 3. The figure shows the percentage of places where the individual has stated that there is homophily in the venue’s social environment for each characteristic. Nightclubs and bars are the category that has the highest homophily, especially in terms of age, restaurants on the other side have the lowest similarity, with income having the lowest share. Cafés show a similar distribution of the social environment to restaurants. Finally, the *I don’t know* answer has a very small percentage of responses (the highest is 8.7% for bars), showing that people are aware of the social environment of the venues they visit and are able to describe it in simple terms.

Assuming that the answers given by the individuals are correct, we have used the information presented above to build the social environment of all venues included in the study. In case of two or more individuals mentioning different social environments for the same venue, we have used both answers as valid to allow for heterogeneity of crowds in time, as venues could attract different groups of individuals on different days. The specific procedure to generate the social environment is the following:

• Age: If the respondent answers that the venue social environment’s age is similar to theirs, we assume the social environment of the place is in a range of ± 5 years to the respondent’s age. For example, if an individual aged 34 mentions that the social environment of restaurant X is similar to theirs, the age of the social environment is between 29 and 39.
• Income: To create the income social environment of the venues, we used a subjective income variable; the question asks about the perceived level of household income compared to the country’s average, with five Likert options from *far below average* to *far above average.* We consider this variable instead of the objective income because the perceived socioeconomic position results from a process of acquiring self-perception and wealth prospects, being related not only to income but also to education and individual and family experiences (Ferreira et al., 2018), therefore individuals with similar household incomes can belong to different socioeconomic groups due to temporal or circumstantial changes in remunerations (i.e., unemployment), different household maintenance costs, or social contexts. If an individual mentions that a venue has a similar socioeconomic social environment, we use its answer to the subjective income question as the income level of the venue’s social environment.
• Cultural origin: This variable was created using the parents’ origin as the variable of interest. The first question asked the respondents if the parents were foreign-born; if the answer is “no,” the person is considered Swiss. If the answer is “yes,” the follow-up question asked what macro-region the parents were originally from. Then, if the person answers that the social environment has a similar cultural origin, the venue has a social environment of the culture specified before. Although the definition of culture has changed with globalization and geographic borders do not necessarily explain cultural similarities anymore (Sycara et al., 2013), the origin country of first- and second-generation immigrants remains a relevant factor in social connections (Galster et al., 2021).

Table 3 explains in detail the variables used in the model. We have excluded temporal and modal restrictions and variables because we are interested in understanding the reasons for choosing one place as part of the individuals’ leisure routine. At the same time, these visits do not necessarily have the same schedule and duration and are not carried out with the same transport mode.

**Table 3.** Venue-level variables used in the choice model.

Variable name	Description
Price	Price level of the venues on a scale from 1 to 3
Age	Age of the individual
High income	Dummy when the household of the individual earns more than 10,000 CHF a month
Rating	Average user rating of the place on a scale from 1 to 5
Attractiveness	Number of other venues within a 4 km radius of the venue of interest
Distance	Logarithm of the Euclidean distance from the individual’s home location to the venue
Homophily age	Dummy variable if the individual has an age in the venues’ social environment range
Homophily income	Dummy variable if the individual belongs to the socioeconomic group of the social environment of the venues
Homophily culture	Dummy variable if the individual shares a similar cultural origin with the social environment of the venues

## Methodology

To estimate the leisure destination choice model, we base our model on the methodology first introduced by Mansky (1977), with a choice set probabilistically integrated into the choice model. The original model proposed by Mansky is as follows:

(1) $$P_{ni}=\underset{C}{\sum }P_{n}\left(i|C\right)\pi_{n}\left(C|i,z\right)$$

In which, *P*_*ni*_ is the probability that individual *n* chooses option *i*, being *C*_*n*_ the evoke set; *P*_*n*_(*i*|*C*_*n*_) is the probability that individual *n* chooses option *i* given *C*_*n*_; and *π*_*n*_(*C*_*n*_|*i*, *z*) is the probability that the choice set of individual *n* is *C*_*n*_ given the observed choice *i* and a vector of variables *z*.

### Choice set selection

As the choice set is unknown, we have to differentiate between the universal set (all alternatives available) and the evoke set (the alternatives that meet certain criteria and are considered by the individual) (Pagliara and Timmermans, 2009). To estimate *π*_*n*_(*C*_*n*_|*i*, *z*) we have generated a choice set with thresholds on the location characteristics (Cantillo and Ortúzar, 2006). The evoke set *C*_*n*_ is:

(2) $$C_{n}=\left\{j|X_{nj}\leq H_{n}\right\}$$

where *X*_*nj*_ is an attribute of the alternative *j* for the individual *n*, *H*_*n*_ is the highest threshold value that the individual *n* considers for the attribute *X*. The defined threshold is 1.5 times the distance from the site that the individual visits for leisure. The value was chosen to reduce the possibility that an unchosen alternative is too far from the individual’s activity space, generating a model that estimates more discerning individuals regarding this specific attribute, avoiding implausible behaviors (Kimya, 2018), such as always choosing the closest venue. The average choice set size is 398.2 with a maximum of 1182. We tested other choice set generation strategies such as importance and strategic samplings, with this spatial restriction strategy being the one with the most reliable results in terms of *rho*^2^, AIC, and BIC.

### Model description

To estimate the probability of choosing an alternative i, following the formulation of McFadden (1973):

(3) $$P_{ni}=\frac{e^{V_{ni}+ln\left(\pi_{n}\left(C_{n}|i,z\right)\right)}}{\sum_{j\in C_{n}}e^{V_{nj}+ln\left(\pi_{n}\left(C_{n}|j,z\right)\right.}}$$

In which, *V*_*ni*_ is the systematic component of the utility of person *n* given by the alternative *i*. As *π*_*n*_(*C*_*n*_|*i*, *z*) includes all alternatives below the threshold *H*_*n*_, and contains *i*, it satisfies the positive conditioning property (if *i*, *j* ∈ *C*_*n*_ and *P*(*i* ∈ *C*_*n*_) > 0, then *P*(*j* ∈ *C*_*n*_) > 0) and the uniform conditioning property (*π*_*i*_(*C*_*n*_|*i*, *z*) = *π*_*i*_(*C*_*n*_|*j*, *z*)), then the probability of the choice set is canceled out. Then, the log-likelihood function is:

(4) $$\begin{array}{c} LL\left(\Omega ,\theta \right)=\overset{N}{\underset{n=1}{\sum }}ln\left(\int_{\beta }\left\{\frac{e^{V_{ni}}}{\sum_{j\in C}e^{V_{nj}}}\right\}f\left(\beta |\Omega \right)d\beta \right) \end{array}$$

(5) $$SLL\left(\Omega ,\theta \right)=\frac{1}{K}\overset{K}{\underset{k=1}{\sum }}\overset{N}{\underset{n=1}{\sum }}ln\left(\frac{1}{R}\overset{R}{\underset{r=1}{\sum }}\left\{\frac{e^{V_{ni}}}{\sum_{j\in C}e^{V_{nj}}}\right\}f\left(\beta |\Omega \right)d\beta \right)$$

with

(6) $$\begin{array}{c} V_{n,i}=f_{ic}^{\mathrm{Price}}\left(x_{n}^{\mathrm{Age}},x_{n}^{\mathrm{Income}}\right)\cdot x_{ic}^{\mathrm{Price}}+\beta^{\mathrm{Rating}}\cdot x_{i}^{\mathrm{Rating}}+\beta^{\mathrm{Att}}\cdot ln\left(x_{i}^{\mathrm{Att}}\right)\\ +\beta^{\mathrm{Dist}}\cdot ln\left(x_{in}^{\mathrm{Dist}}\right)\;+\beta_{nh}^{\mathrm{Homophily}}\cdot x_{\mathrm{inh}}^{\mathrm{Homophily}} \end{array}$$

and,

(7) $$\begin{array}{ll} f_{ic}^{\mathrm{Price}} & =\left(\beta_{ic}^{\mathrm{Price}}+\beta_{ic}^{\mathrm{Price,Age}}\cdot x_{n}^{\mathrm{Age}}+\beta_{ic}^{\mathrm{Price,Income}}\cdot x_{n}^{\mathrm{Income}}\right) \end{array}$$

(8) $$\begin{array}{cc} \beta_{nh}^{\mathrm{Homophily}} & =exp\left(\mu_{ln\left(\beta_{h}^{\mathrm{Homophily}}\right)}+\sigma_{ln\left(\beta_{nh}^{\mathrm{Homophily}}\right)}\cdot r_{N}\right) \end{array}$$

where *β* is the taste coefficient of each variable and *x* is the vector of attributes for individual *n* at venue *i*, when applicable. Price has a subindex *c* as it has three levels from 1 to 3, while homophily subindex h represents the three homophilic preferences studied. The scale parameter has a mean value $\mu_{ln\left(\beta_{h}^{\mathrm{Homophily}}\right)}$ , a standard deviation $\sigma_{ln\left(\beta_{n,h}^{\mathrm{Homophily}}\right)}$ , and a log-normal distributed individual-specific random component *r*_*N*_. We tested different distributions of taste heterogeneity, with the log-normal fitting best to the data. The socioeconomic variables included, as interactions with price, were age and high income, income has been included as a dummy because it had the highest explanatory power compared to other specifications. Both sociodemographic variables are due to their expected impact on price level preferences, since venues with higher prices also have better expected services, we were expecting older and wealthier individuals to have higher preferences for these venues. The model was estimated using the Apollo R package (Hess and Palma, 2019), the BGW algorithm (Bunch et al., 1993), with 1000 Halton draws.

## Model results

### Multinomial logit

Before the mixed model, we have estimated a model with $\beta_{n,h}^{\mathrm{Homophily}}=\mu_{\beta_{h}^{\mathrm{Homophily}}}$ . The results of the food venues and nightlife models are presented in Table 4. For the food services models, the control variables show expected signs; the estimates for rating and attractiveness are positive, while the estimates for price and distance are negative. The interaction terms between price and the socioeconomic variables are positive (except price = 2 and its interactions that are non-statistically significant), showing that older and wealthier individuals prefer more expensive venues than younger and lower-income individuals. The nightlife venues show similar results, but income being non-statistically significant in any of the price levels. Compared with food services, we see that age has a higher impact on the preference for more expensive nightlife venues, while income is more relevant for the food services’ price preference. Both models show negative estimates regarding distance, with nightlife venues having a lower preference for closer venues. In terms of attractiveness, the estimate for nightlife venues is more than double the estimate for food venues; this could be related to the interest of individuals in visiting nightlife venues in more vibrant parts of the city.

**Table 4.** MNL model of homophily in destination choice.

	Food services			Nightlife		
	Estimate	s.e.	*p*-value	Estimate	s.e.	*p*-value
Price = 2	−0.53	0.70	0.22	−0.63	1.15	0.29
Price = 3	−1.29	0.90	0.08	−2.26	1.49	0.06
Distance	−1.00	0.03	0.00	−0.95	0.07	0.00
Rating	0.26	0.07	0.00	0.42	0.10	0.00
Attractiveness	0.09	0.01	0.00	0.21	0.03	0.00
Age * price = 2	0.01	0.01	0.14	0.03	0.03	0.09
Age * price = 3	0.02	0.02	0.09	0.06	0.03	0.02
Income * price = 2	0.12	0.28	0.33	−0.24	0.68	0.36
Income * price = 3	0.54	0.34	0.06	0.03	0.74	0.48
Age homophily	1.15	0.08	0.00	1.33	0.09	0.00
Income homophily	0.69	0.08	0.00	0.36	0.10	0.00
Culture homophily	0.60	0.08	0.00	1.13	0.10	0.00
Rho-squared	0.11			0.14		
AIC	17,738.7			7210.73		
BIC	17,805.6			7268.84		

In terms of the preference for homophily, the nightlife venues model, on average, has higher estimates. In both models, age homophily is the most important variable, with estimates of 1.15 and 1.33, respectively. The second most important homophily variable for food venues is income, with an estimate of 0.69, followed by cultural origin with an estimate of 0.60. For nightlife venues, homophily in cultural origin is more important than income homophily with estimates of 1.13 and 0.36, respectively.

### Mixed multinomial logit

After estimating the multinomial logit model, we have included the variables to measure the taste heterogeneity. The results are presented in Table 5. The goodness of fit of the mixed model presents an improvement in *ρ*^2^ and slight improvements in AIC and BIC. Regarding the results, the mixed model of the food venues shows estimates similar to the MNL model, except for the estimate of price = 3, which is slightly more negative. In the nightlife model, the value of the estimate of price = 2 is reduced from −0.63 to −1.37, the estimate of income * price = 2 goes from −0.24 to −0.04, and the estimate of income * price = 3 changes from −0.03 to 0.26, but this estimate is not statistically significant.

**Table 5.** Mixed MNL model with heterogeneity in preferences for homophily in destination choice.

	Food venues			Nightlife venues		
	Estimate	s.e.	*p*-value	Estimate	s.e.	*p*-value
Price = 2	−0.57	0.72	0.22	−1.37	1.17	0.12
Price = 3	−1.47	0.96	0.06	−3.25	1.61	0.02
Distance	−1.04	0.03	0.00	−0.94	0.08	0.00
Rating	0.25	0.07	0.00	0.43	0.10	0.00
Attractiveness	0.09	0.01	0.00	0.20	0.02	0.00
Age * price = 2	0.02	0.01	0.12	0.05	0.03	0.04
Age * price = 3	0.03	0.02	0.07	0.08	0.03	0.01
Income * price = 2	0.12	0.29	0.33	−0.04	0.75	0.48
Income * price = 3	0.54	0.36	0.07	0.26	0.83	0.38
*μ* age	−0.35	0.14	0.00	−0.03	0.13	0.40
*μ* income	−1.69	0.42	0.00	−6.40	3.04	0.02
*μ* culture	−1.38	0.33	0.00	−0.24	0.19	0.11
*σ* age	−1.00	0.11	0.00	−0.93	0.15	0.00
*σ* income	−1.91	0.36	0.00	−4.65	1.67	0.00
*σ* culture	−1.49	0.24	0.00	−1.30	0.26	0.00
Rho-squared	0.13			0.15		
AIC	17,427			6816		
BIC	17,511			6888		

Due to the interactions that the price attribute has in the model, Table 6 presents a comprehensive examination of the utilities of each demographic group in terms of preferences for price levels. As explained above, older and wealthier individuals tend to have preferences for more expensive venues; more specifically, low-income individuals have an average negative preference for venues with price = 2 (compared to price = 1) for all age groups except for 40 and older, who have a positive preference for venues with price = 2. Conversely, high-income individuals over 30 prefer food venues with a price level = 2. Low-income individuals under 60 years of age have a negative preference for food venues with a price level = 3. In contrast, high-income individuals older than 40 prefer these venues over cheaper venues. While for nightlife venues, even if the estimate for price = 2 is negative, only 20-year-olds have a negative preference for this type of venue, as older individuals have a positive preference for venues with price level = 2. For nightlife venues with price = 3, individuals have a positive preference when they are 40 years or older. Finally, we have excluded the income variable from nightlife venues because, as shown in the Table 5, the income of the individual does not play a role in the preference for nightlife venues. Changes in preferences, from negative to positive, are due to the price level being a category related to the service offered, as well as the price. Concerning the variables of interest, the value *exp*(*μ*) provides the mean preference for each homophily estimate, while *exp*(*σ*) measures the heterogeneity of the preferences. The combination of both estimates provides the range of preferences for each of the variables of interest, which is assumed to be log-normal.

**Table 6.** Utilities of the socioeconomic groups for the price level of the venues.

	Age	20	30	40	50	60
Food venues						
Price = 2	Low income	−0.26	−0.11	0.04	0.19	0.34
	High income	−0.14	0.01	0.16	0.31	0.47
Price = 3	Low income	−0.96	−0.70	−0.45	−0.20	0.06
	High income	−0.42	−0.17	0.09	0.34	0.59
Nightlife venues^a^						
Price = 2		−0.36	0.15	0.65	1.16	1.66
Price = 3		−1.62	−0.80	0.01	0.83	1.65

^a^Income has been excluded of the nightlife table as it shows no statistical significance in any of the interactions with price.

### Willingness to travel

To make an easier comparison of the different values of the preference for homophily in the social environment, we have estimated the willingness to travel (WTT) indicators calculated from the mean preference attribute for each homophily preference, presented at Table 7; this value measures how much extra the average individual is willing to travel to be with similar others. These indicators allow to directly compare the preference coefficients and measuring the trade-off between distance and social environment, models, and venues. Comparing the MNL and Mixed MNL model, in the latter model, all willingness to travel is relatively smaller than in the former model, due to the distribution of the taste heterogeneity, with cultural origin in food services and income in nightlife venues the most notable changes. When comparing venue categories, nightlife has a higher average willingness to travel, in terms of preferences for age homophily, and the WTT of the nightlife venues is 1.02 versus 0.68. For food services, the average individual is willing to travel 0.18 times the distance to be in a social environment with similar income. At the same time, this attribute is negligible for nightlife venues (0.00). Finally, cultural origin has a lower willingness to travel for food services compared to nightlife venues (0.24 vs 0.84 extra distance).

**Table 7.** Homophily-distance willingness to travel, measured as increase in percentual distance.

	Food venues		Nightlife venues	
	MNL	Mixed MNL	MNL	Mixed MNL
	$\frac{\beta_{h}}{\beta_{d}}$	$\frac{\exp\left({\bar{\mu }}_{h}\right)}{\beta_{d}}$	$\frac{\beta_{h}}{\beta_{d}}$	$\frac{\exp\left({\bar{\mu }}_{h}\right)}{\beta_{d}}$
Age homophily	1.15	0.68	1.41	1.02
Income homophily	0.69	0.18	0.38	0.00
Culture homophily	0.60	0.24	1.19	0.84

^a^As the WTT depends on distance, the willingness to travel is estimated by multiplying these values by $x_{i,n}^{\mathrm{Dist}}$ .

**Figure 4.**
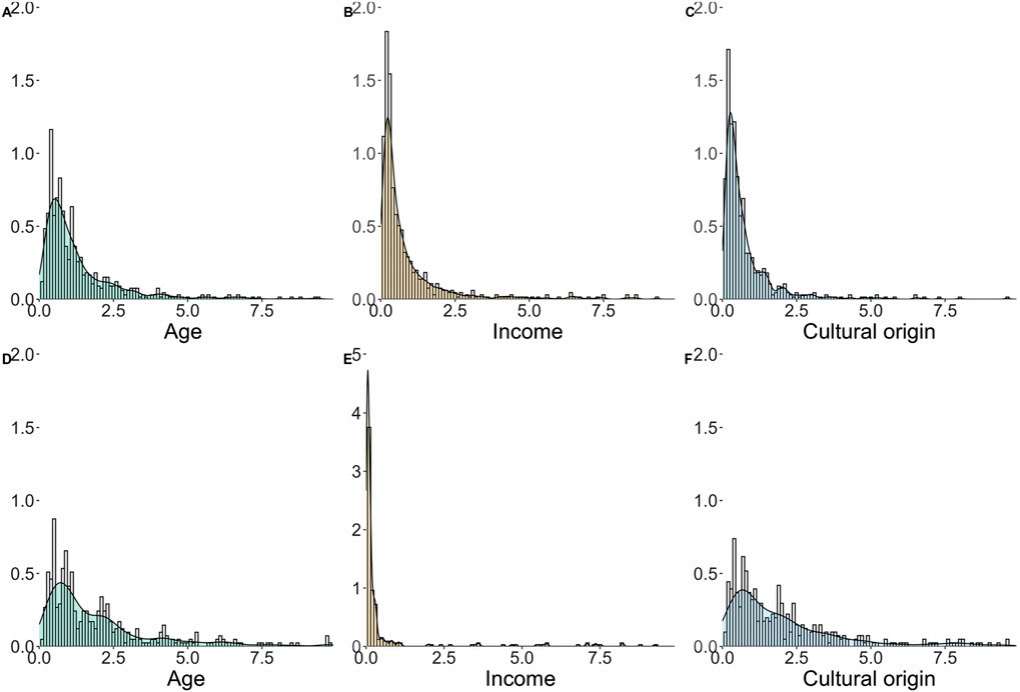
Conditional (bars) and unconditional (density) distributions of willingness to travel. (A, B, and C) Food venues and (D, E, and F) nightlife venues. Note: y-axis of graph E uses a different scale.

A visual representation of the heterogeneity of the WTT estimates is shown in Figure 4, where the conditional (bars) and unconditional (density) distributions of the three preferences for homophily are presented by model. Regarding food services, cultural origin and income have high peaks, showing greater homogeneity in preferences compared to age homophily. In the case of the nightlife venues model, age and cultural origin homophily show a similar distribution of the conditionals, with both distributions having a high heterogeneity, while the preference for income homophily is very close to zero for most of the individuals, which explains the low mean preference shown above.

## Discussion and conclusion

We have analyzed the importance of the social environment in destination choice, measured as the preference for homophily of three characteristics of the social environment: age, income, and cultural origin. The two models, food venues and nightlife venues, have shown a positive and significant impact of the three socioeconomic homophilic preferences when choosing a venue to perform leisure, age homophily being the most relevant variable in both models. In contrast, income homophily is relevant, but with a low WTT, when choosing a restaurant or cafe, whereas its importance is negligible when choosing nightlife venues, which coincides with a statistical insignificance of the income interaction with price in those venues; homophily of cultural origin shows higher relevance when choosing bars or nightclubs than when choosing food venues. The difference in homophilic preferences shows that individuals have different preferences for the social environment depending on the context of the social activity. The higher preferences for homophily in nightlife venues can be related to the social expectations of the activity: bars and nightclubs tend to have more interaction between unknown individuals, and they are better venues to potentially meet new people, while restaurants and cafés depend more on clique interaction than interactions “between tables.” These distinct preferences are consistent with previous research on social networks showing that homophilic preferences have baseline patterns with differences depending on the type of relationship formed between individuals (McPherson et al., 2001). The distribution of the preferences for homophily also depends on the type of venues; preference for income homophily has a higher variance in food venues than in nightlife venues, while the opposite happens with age and cultural origin, which shows that the distribution of preferences in food services tends to be more homogeneous than in nightlife venues. The low WTT of income homophily in both types of services could be related to inequality not being a recurrent problem in Switzerland, which has had stable inequality indicators since 1930 (Institut für Schweizer Wirtschaftspolitik, 2024), while it has increased significantly since 1980 in most countries (Chancel et al., 2022). On the other hand, there has been a growing problem of racial profiling in recent years, which could be related to the preferences for homophily in cultural origin.^1^

The study makes two contributions to the literature. First, the measurement of homophilic preferences in leisure activities; homophily is an essential aspect of social networks and can be observed in many types of social interaction, but as far as the authors are aware, this is the first time it has been measured as a characteristic that affects the choice of the specific venue to perform leisure activities. The results can help understand how individuals choose where to share common spaces with unknown individuals, these interactions are an essential aspect of urban social life as they generate a sense of community and belonging, which could result in socialization and the formation of friendship ties; in practice, with these results new public policy can be implemented in order to create more welcoming urban leisure spaces, that integrate different groups of society on a daily basis. Previous studies have found that some areas of the city tend to gather individuals from different social backgrounds, but visiting the same areas does not necessarily mean that they perform leisure activities in the same venues. Therefore, more research is needed to understand the origins and measure leisure segregation and define whether segregation can still occur in physical proximity; this is a first approach to this task.

The second contribution is to show the potential of using venue-level information to estimate models of leisure destination choice. In this paper, we have mixed survey data with social network services (SNS) data to include three types of characteristics: individual level (age and income), venue level (category, price, area attractiveness, and rating), and individual venue level (distance and homophily). The use of venue-level characteristics for leisure destination choice is a methodological advancement in comparison to previous models that analyze meso-destination characteristics, such as demographics and agglomeration effects, because using venue characteristics such as price or rating can increase the number of options available for individuals as well as make comparisons between spatially contiguous venues possible, increasing the level of details the model can provide.

The literature on homophily has shown that preferences lead to network segregation, even when preferences are minor (Goodreau et al., 2009; Hatna and Benenson, 2015; Schelling, 1971); for this reason, it is relevant to understand the preferences of individuals for the social environment of their leisure venues. However, a second potential venue characteristic in leisure activities can lead to age and income segregation: the price category. The preference for price category varies depending on age and income, with the sign of the parameters varying from negative to positive as individuals get older and wealthier; this could generate higher levels of segregation in leisure activities than if only homophilic preferences were to be considered.

We want to point out two main limitations of this study. The first is how the social environment is defined, as collecting data on the social environment for more than 1500 venues is resource intensive; we had to rely on the information provided by the respondents, which can be prone to measurement errors. The second limitation is the number of socioeconomic characteristics used for homophilic preferences; homophily is a complex phenomenon that depends not only on the socioeconomic characteristics and interests of the individual but on shared values, beliefs, or a multifactorial combination of all these variables (Block and Grund, 2014). Additionally, the data has been collected in Zurich, and therefore the preferences estimated are not generalizable to other locations. However, this study has shown the importance of the three homophilic preferences studied in the individual’s process of leisure destination choice, and it is a first step in understanding how individual preferences can generate segregation in daily leisure activities.

## Data Availability

The data set is available on demand; please contact the corresponding author.*

## Note

1. Source: Swissinfo.ch.
